# Consensus Statements among European Sleep Surgery Experts on Snoring and Obstructive Sleep Apnea: Part 1 Definitions and Diagnosis

**DOI:** 10.3390/jcm13020502

**Published:** 2024-01-16

**Authors:** Ewa Olszewska, Andrea De Vito, Peter Baptista, Clemens Heiser, Carlos O’Connor-Reina, Bhik Kotecha, Olivier Vanderveken, Claudio Vicini

**Affiliations:** 1 Department of Otolaryngology, Sleep Apnea Surgery Center, Medical University of Bialystok, 15-276 Bialystok, Poland; 2 Department of Surgery, Morgagni-Pierantoni Hospital, Health Local Agency of Romagna, 47121 Forlì, Italy; dr.andrea.devito@gmail.com; 3 Clinica Universidad da Navarra, Departmento de Orl, 31008 Pamplona, Spain; peterbaptista@gmail.com; 4 Faculty of Medicine and Health Sciences, University of Antwerp, 2000 Antwerp, Belgium; hno@heiser-online.com (C.H.); olivier.vanderveken@uza.be (O.V.); 5 Department of Otorhinolaryngology/Head and Neck Surgery, Klinikum Rechts der Isar, Technical University of Munich, 80333 Munich, Germany; 6 Hospitales Quironsalud Marbella, 29603 Malaga, Spain; carlos.oconnor@quironsalud.es; 7 Nuffield Health Brentwood, Essex, Brentwood CM15 8EH, UK; bhikkot@aol.com; 8 UME Health, 17 Harley Street, London W1G 9QH, UK; 9 Department of Otorhinolaryngology, Head and Neck Surgery, Antwerp University Hospital, 2650 Antwerp, Belgium; 10 GVM Care & Research ENT Consultant, GVM Primus Medica Center, GVM San Pier Damiano Hospital, 48018 Faenza, Italy; claudio@claudiovicini.com

**Keywords:** snoring, sleep apnea, obstructive sleep apnea, consensus, definitions, diagnosis, Delphi method

## Abstract

Seeking consensus on definitions and diagnosis of snoring and obstructive sleep apnea (OSA) among sleep surgeons is important, particularly in this relatively new field with variability in knowledge and practices. A set of statements was developed based on the literature and circulated among eight panel members of European experts, utilizing the Delphi method. Responses in agreement and disagreement on each statement and the comments were used to assess the level of consensus and develop a revised version. The new version with the level of consensus and anonymized comments was sent to each panel member as the second round. This was repeated a total of five rounds. The total number of statements included in the initial set was 112. In the first round, of all eight panelists, the percentage of questions that had consensus among the eight, seven, and six panelists were 45%, 4.5%, and 7.1%, respectively. In the final set of statements consisting of 99, the percentage of questions that had consensus among the 8, 7, and 6 panelists went up to 66.7%, 24.2%, and 6.1%, respectively. Delphi’s method demonstrated an efficient method of interaction among experts and the establishment of consensus on a specific set of statements.

## 1. Introduction

Snoring is the primary nocturnal symptom of obstructive sleep apnea (OSA) syndrome. Its occurrence usually prompts direct questioning about other symptoms of OSA, including breathing pauses during the night, unrefreshing sleep, and daytime sleepiness. Snoring is a very common finding, with a 38–76% prevalence in different populations [1,2,3]. In the presence of snoring in a setting of OSA-related cardiovascular and metabolic comorbidities, the necessity for further work-up is even more precious.

OSA is the most common sleep-disordered breathing (SDB) in adults [4]. The overall prevalence of OSA ranges between 4% to 38% [5,6,7] in some cohorts, which is expected to rise shortly due to the recent increase in obesity [7,8].

OSA is characterized by recurrent episodes of partial or complete upper airway collapse during sleep [9]. The resulting reduction in airflow often leads to acute gas exchange disorders with recurrent sleep arousals. The impacts on the quality of life of untreated OSA patients are numerous and include excessive daytime sleepiness, impaired work performance, cognitive dysfunction, and various impaired health parameters. Observational and experimental evidence also suggests that untreated OSA contributes to the development of systemic hypertension, cardiovascular disease, abnormalities in glucose metabolism, and stroke [10,11,12,13,14,15,16,17]. Early recognition and adequate treatment can diminish the neurocognitive consequences and improve cardiovascular health [18].

The pathogenesis of OSA is multifactorial, involving several complex mechanisms [19]. These include the anatomical configuration of the upper airway and its dynamic alterations, the efficacy of the dilator muscles in preserving airway patency, and the stability of respiratory control systems. Crucial to this latter aspect is the respiratory excitation threshold, which governs arousal responses, and the respiratory drive, commonly referred to as loop gain. Each of these factors contributes significantly to the onset and progression of OSA, underscoring the condition’s multifaceted nature [19,20].

Given this complexity and heterogeneity, OSA is a challenge for the clinicians involved in its evaluation and management. Multiple disciplines are involved in diagnosing and treating OSA, such as pulmonology, otolaryngology, head and neck surgery, neurology, sleep medicine, oral-maxillofacial surgery, dentistry, anesthesiology, speech therapy, psychiatry, cardiology, sleep physiology, nursing staff, sleep lab technicians, etc., leading to differences in definitions and approaches.

Each country and region may have differences in health standards, socio-economic conditions, culture, social and health priorities, knowledge, and awareness about snoring and OSA as a problem and the need for diagnosis and treatment.

Several consensus statements and guidelines addressing the evaluation and management of adult patients with OSA have been published in the last 30 years throughout the world and include, among others, the Adult Obstructive Sleep Apnea Task Force of the American Academy of Sleep Medicine, 2009 [21]; Irish Sleep Society, 2015 [22]; European Respiratory Society, 2021 [23]; and NICE, 2021 [24]. These guidelines consider different aspects such as pathophysiology, clinical evaluation, subjective and objective diagnostic tests, and diverse treatment modalities.

A recent international consensus statement on obstructive sleep apnea (ICS: OSA) [25] was created to summarize available evidence into a format allowing clinicians to examine diagnosis and management options for adult OSA. The main objective of this ICS on OSA was to summarize and consolidate the available knowledge on the diagnosis and treatment of OSA. However, it did not elaborate on specific indications of surgical treatments in detail.

Therefore, we established a panel of otolaryngology/head and neck surgery experts in snoring and OSA to develop statements on diagnosing and treating snoring and OSA in adults. We aimed to reach a consensus. In the first phase, the expert panel explored an agreement on definitions, patient criteria for diagnosis, and clinical evaluation of snoring and OSA.

This panel’s initial statements included general comments regarding snoring and sleep apnea. Further statements focused on surgical treatment and on various aspects of palatal surgery for snoring and OSA. There will be future reports on these statements which will include the decision making for surgery for snoring and OSA, peri-operative considerations, palate surgery, outcomes, follow-up, complications, and postoperative management.

## 2. Materials and Methods

To develop consensus statements among European experts on the diagnosis of Snoring and OSA, an initiative was launched to engage experts in the field. A structured approach was proposed, incorporating the modified Delphi method, to determine the level of consensus [26,27,28,29]. A thorough literature review was conducted by the first author (EO), creating an initial draft of the consensus statements. These statements were systematically categorized into sections such as definitions, patient criteria, diagnosis, considerations for surgical intervention, pre-operative considerations, procedures related to palatal surgery, post-operative outcomes and monitoring, and potential complications.

After collective review and discussion, the initial set of statements was solidified by the core group of three colleagues. The foundational team pinpointed esteemed otolaryngologists with expertise in snoring and OSA management, emphasizing palatoplasty as the primary surgical point of consensus. Consequently, a panel comprising 8 experts was formed through invitation.

Panelists were provided an Excel spreadsheet containing the statements. They were prompted to indicate their agreement or disagreement with each statement and to provide comments or suggest modifications where necessary. After collecting all feedback, the results were aggregated, highlighting the count and percentage of panelists agreeing or disagreeing with each statement and calculated.

In alignment with the predefined research methodology, the primary author undertook an in-depth analysis of the feedback, paying particular attention to both the quantitative responses and the qualitative comments. Through this meticulous review, certain issues and inconsistencies that might have impacted the panelists’ feedback were identified. Given these observations, the research team deemed it necessary to draft a refined set of statements, which would serve as the basis for the second-round survey instrument.

This revised document was systematically organized to ensure clarity and precision. Each modified statement was clearly marked to distinguish the changes from the original, ensuring transparency in the modifications made. Additionally, to provide a comprehensive overview, summary metrics accompanied each statement. These metrics detailed the number of panelists who had responded to a specific statement, the count of those who agreed, and the count of those in dissent. Furthermore, a percentage consensus was computed not just for each statement but also as an aggregate for the entire set of statements.

Moreover, to ensure anonymity yet facilitate understanding, comments related to specific statements, as provided by the panelists during the initial round, were incorporated without attributions. This was instrumental in offering a holistic view of the collective sentiment of the panel without compromising the confidentiality of individual responses.

The document was then tailored for each panelist. Their individual files incorporated their previously recorded responses juxtaposed with options to either “agree” or “disagree” for the revised statements. An additional column was integrated to allow for any new feedback, suggestions, or concerns that the panelists might wish to share considering the modified statements.

These personalized files were disseminated to each panel member after this systematic reconfiguration. The communication underscored the importance of their role in the iterative process and solicited their invaluable input on the modified set of statements, ensuring a robust and comprehensive understanding of the subject matter.

A second-round iteration was initiated in alignment with the modified Delphi method, aiming for a minimum of 75% consensus. Adjustments were made to the statements based on panelists’ feedback and alterations were distinctly marked. The revised document presented the degree of consensus both numerically and as a percentage and included anonymized comments from the panelists. Each participant was given their prior responses, with the opportunity to modify their feedback, offer fresh comments, or propose further refinements to the statements. Customized second-round files, reflecting these elements, were disseminated to each panel member.

After collecting feedback from the second round, subsequent third and fourth rounds of statement evaluations were undertaken using a similar methodology. Each round saw the creation and dissemination of updated statement files to the panelists, reflecting the cumulative feedback and modifications from the preceding rounds.

Following the fourth round, a conclusive verification statement file was generated. Within this file, responses that represented the minority stance among the 8 panelists (those viewpoints held by only 1, 2, or 3 members) were distinctly highlighted. This design was intentional, providing each panelist with the opportunity to re-evaluate and reaffirm their stance, especially when it contrasted with the majority viewpoint. To ensure an impartial evaluation and uphold the integrity of the process, the specific identities of the panelists whether in agreement or dissent with a given statement were concealed. This anonymity empowered each panel member to solidify their position and, if deemed necessary, furnish a rationale for their viewpoint.

Following the established research protocol, subsequent data collection and evaluation rounds were again meticulously undertaken. Specifically, for both the third and fourth iterations, there was a comprehensive process of gathering panelists’ feedback, systematically reviewing their responses, quantifying the degree of consensus, and scrutinizing the qualitative feedback in the form of comments and suggestions. Having attained this further analysis, a renewed set of files was judiciously prepared and subsequently dispatched to the panelists to solicit further input.

For the culmination of the study, a definitive round of verification files was contrived. This penultimate file did more than merely encapsulate the responses accrued during the fourth round; it was designed with an analytical lens to discern the nuances of panelist agreement and disagreement. To enhance clarity and facilitate further introspection, responses within these individualized files were distinctly marked if a panelist’s perspective diverged significantly from the majority. Such highlighting was mainly employed if a panelist’s view was representative of a smaller subset, specifically 1 to 3 panelists out of the total of 8, thereby illustrating a lack of consensus with the predominant view.

Upon collecting feedback from the verification files, a definitive set of statements was consolidated. Subsequently, a summarized representation, detailing the consensus percentage for each statement, was disseminated to all panel members. This summary ensured clarity by presenting an aggregate view, deliberately omitting individual panelist stances.

Upon collating and analyzing these highlighted responses, the research team was equipped to finalize and present the concluding set of statements, which forms the crux of this study.

The first author then articulated a strategy to draft the initial manuscript, spotlighting statements that delineated the definitions and diagnostic criteria for snoring and OSA. Following endorsement of this plan, EO embarked on the drafting phase, meticulously crafting each segment of the manuscript. These segments were sequentially circulated among the panel members, ensuring each section was vetted iteratively.

**Figure 1:**
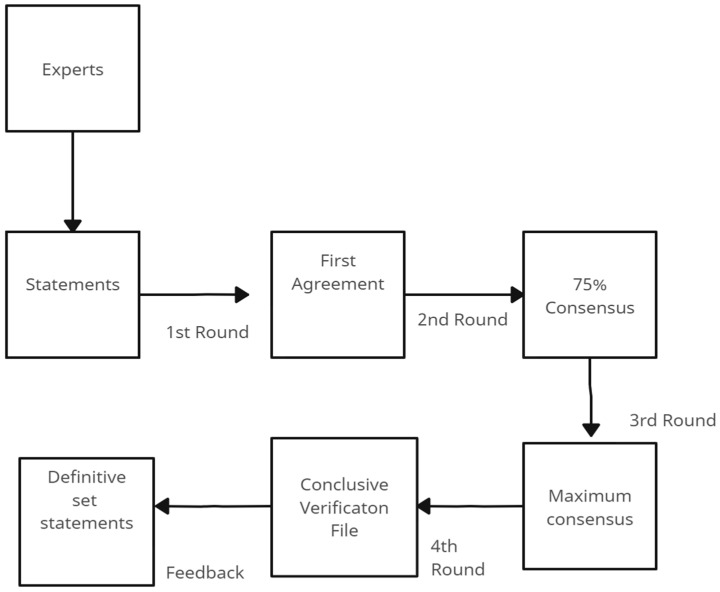
Flow chart of the revision cycle of statements.

After each section underwent a preliminary review and requisite modifications, the comprehensive manuscript, tailored to the specifications of the intended journal, was pieced together. This consolidated draft was then disseminated to all panelists, inviting them to conduct a thorough review, suggest refinements, and ultimately grant their endorsement before the document proceeded to submission (See Figure 1).

## 3. Results

In a comprehensive examination of the consensus regarding topics related to definitions, patient criteria, and diagnosis concerning scoring and OSA, an initial set comprising 112 statements was disseminated to an expert panel. This panel comprised eight members, evaluated each statement, and marked them either in concurrence or in dissent.

In the primary phase of evaluation, the panelists registered their acquiescence or disagreement with an average of 72.4% of the disseminated statements. Notably, there was an absence of response for 27.6% of the available statement options across all eight panelists. In the first round, 46.4% of the statements achieved consensus by the full panel, 13.4% by seven panelists (87.5% representation), and 12.5% by six panelists (75% representation). By the conclusion of the initial survey, a consensus of at least six panelists was established for 72.3% of the presented statements.

The terminal set of statements, oriented around the pivotal themes of definitions, patient criteria, and the intricate dynamics of scoring related to OSA, comprised a total of 99 assertions. An intricate examination of this set reveals that 62 of these statements were autonomous in nature, whilst a subset of 15 statements stood as overarching themes, each further bifurcated into specific sub-statements, totaling 37 in number. These sub-statements, delineated as “a”, “b”, “c”, and so forth, were intended to expound on the multifaceted aspects and intricacies of their respective parent statements.

**Figure 2:**
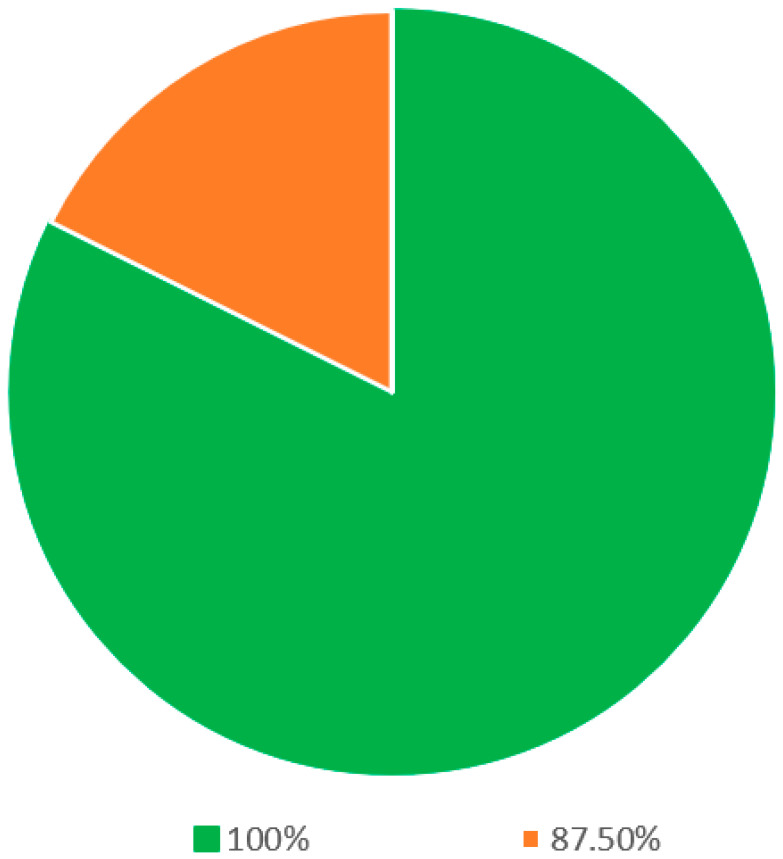
Statements on the definitions regarding snoring and OSA.

Within the segment delineated as definitions (as elucidated in Table 1), 17 statements were presented for evaluation. Following rigorous deliberation in the final round, a commendable consensus was achieved among all eight panelists for a significant subset of these statements. Precisely, 14 out of the 17 statements, equivalent to 82.4% of this section, witnessed unanimous agreement (Figure 2). The statements that garnered this overarching consensus encompassed the terminological definitions of critical concepts such as sleep, snoring, sleep apnea, sleep-disordered breathing, OSA, hypopnea, mixed apnea, the apnea–hypopnea index (AHI), positional obstructive sleep apnea (POSA), rapid eye movement (REM) sleep, sleep quality, sleep study, polysomnography, and home sleep study. In the remaining three statements, seven out of eight panelists reached a consensus.

**Figure 3:**
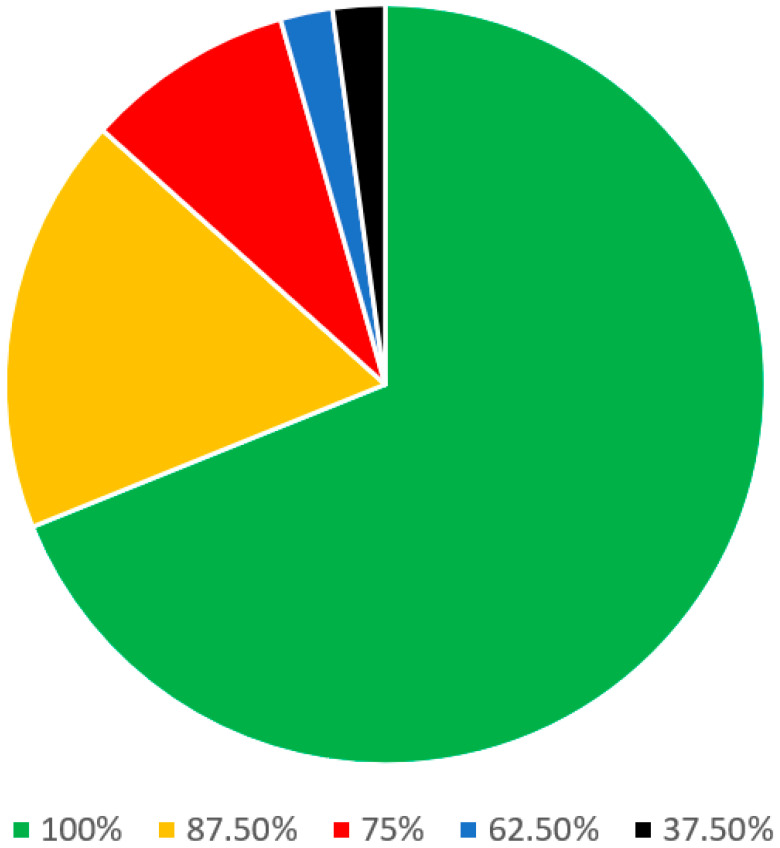
Statements on the patient criteria regarding the diagnosis of snoring and OSA.

In the patient criteria regarding the diagnosis of snoring and OSA section (Table 2), there were a total of 46 statements. Of these, 25 statements were stand-alone and 9 parent statements had 21 sub-statements. A consensus was reached among all 8 panel members on 31 out of 46 (67.4%) statements. On 9 other statements (19.6%), there was agreement among the 7 panelists. Regarding the remaining 4 statements (8.7%), there was agreement among the 6 panelists (Figure 3).

**Figure 4:**
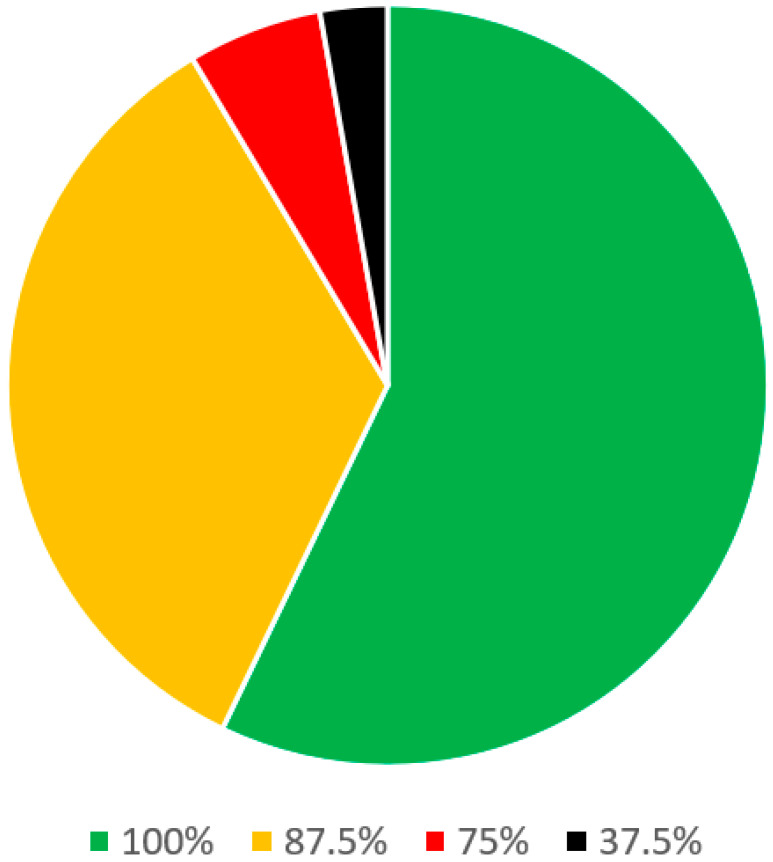
Statements on clinical evaluation of snoring and OSA.

In the clinical evaluation of snoring and OSA section (Table 3), there were 36 statements. Of these, 20 statements were stand-alone and 6 parent statements had 16 sub-statements. Of these, a consensus was reached among all 8 panel members on 21 (58.3%) statements. On 12 statements (33.3%), there was agreement among the 7 panelists and on 2 remaining statements (5.6%) there was agreement among the 6 panelists (Figure 4).

## 4. Discussion

Many medical disciplines, such as pulmonology, neurology, ENT, dentistry, etc., take care of and study OSA patients. A few multidisciplinary institutions have an objective shared clinical approach to OSA patients, whereas, in a single institute, one discipline often has the lead over the complete clinical and/or therapeutical management of OSA patients. Considering the significant difference in OSA definitions, diagnostic and treatment selection criteria, and disagreement between the medical disciplines regarding treatment options, it is not possible at the moment to achieve a universally accepted consensus on all the OSA disease aspects. Recently, an international consensus statement on OSA attempted to identify guidelines in the clinical–therapeutic aspects of OSA, bringing together some of the leading experts in the field [25], highlighting the importance of opinion leaders, with significant experience in the field of SDB, in identifying acceptable definition, diagnostic, and therapeutical protocols valid for the practitioners in daily practice.

Moreover, there are considerable differences among otolaryngologists regarding the indications and surgical techniques for the management of snoring and OSA. For instance, the uvulopalatopharyngoplasty (UPPP) was first introduced by Ikematsu in 1964 to be applied in simple snoring patients. In 1981, Fujita was the first to apply UPPP in OSA patients [30]. Since then, several modifications to the traditional UPPP have been introduced, moving from partial demolition procedures that act mainly on the median portion of the soft palate to procedures that have the aim of stiffening the soft palate, acting also to remodeling the lateral wall pharyngeal muscles [31,32]. Currently, there is no consensus about the proper surgical technique for each OSA patient. Ongoing uncertainty in the fundamental understanding of OSA pathophysiology significantly contributes to the observed heterogeneity in study results [33].

This paper reports on the effort of a group of European experts in the field of sleep surgery to discuss statements on terminology and diagnostic processes related to snoring and OSA. The goal was to explore the level of consensus among the experts from various European countries with somewhat similar SDB prevalence, risk factors, education, socio-economic conditions, and availability of diagnostic tools, compared to the rest of the world. On the other hand, this effort is expected to reflect differences among the European countries.

To collect the experts’ opinions, the modified Delphi method uses panel members’ collective opinions, universally accepted as a systematic and structured process, to develop consensus statements. The main advantage of the Delphi method and its modification is the anonymity of individual panelists, which avoids the potential bias of dominance or group conformity, often reported in face-to-face group meetings [34,35]. Another advantage of the modified Delphi method is the ease of changes in the proposed statements; when needed for various reasons, differences among the panelists are experienced.

In the first round of the present study, an overall low percentage (72.3%) of the statements were answered because ambiguity in their interpretation could characterize some statements or there was an overlap of content or meaning in some statements, there was a lack of coverage or meaning in the first statements’ round and, finally, the instructions regarding how to fill out the responses were considered not completely clear.

Using the modified Delphi method, it has been possible to change and clarify all the statements with misunderstandings, reaching an overall high percentage (87.5–100%) of agreement in all three sections of the consensus: definitions regarding snoring and OSA, patient criteria regarding the diagnosis of snoring and OSA treatment, and clinical evaluation of snoring and OSA.

In the snoring and OSA definition section, the eight panelists reached 100% agreement in 14 out of 17 statements. All the sleep events definitions (Statements 1–12) reached 100% of the agreement, except for the definition of an apnea event (Table 1, Statement 6), which obtained 87.5%. The reason could be related to the different descriptive vs. polygraphy apnea definitions. The polygraphy definition of apnea reported by the American Academy of Sleep Medicine (AASM) [36] stated that apnea is intended a drop in peak signal excursion by >90% of pre-event baseline for >10 s, using an oronasal thermal signal, whereas a descriptive definition, also reported in AASM official documents, stated that apnea is a breathing disorder that involves a complete cessation of airflow, despite an ongoing effort to breath. One definition does not deny the other.

The definition of sleep quality and the overall classification of sleep studies achieved 100% agreement. Otherwise, another statement of incomplete agreement was about the non-REM sleep stage definition (Statement 12). Finally, a disagreement was found about the polygraphy definition (Statement 16).

In the patient criteria regarding the diagnosis of snoring and OSA, the eight panelists reached an agreement in 22 out of 25 stand-alone statements. The first statement of disagreement (87.5%; Statement 6 Table 2) is about the subjective methods for the loudness measurement of snoring, such as the visual analog scale method, considering that in most cases, this parameter is reported by the bed partner, introducing several biases for the reliability of this parameter in this way. Until now, there are no officially reported methods for the snoring loudness measures but, since snoring is one of the first and most cardinal nocturnal symptoms of OSA, its presence should direct medical history about other symptoms of OSA, including breathing pauses at night, snorting, gasping phenomenon, unrefreshing sleep, and excessive daytime sleepiness, especially when the presence of snoring is identified in a potential patient with OSA-related comorbidities [25].

Another statement that reached 87.5% agreement is related to gastroesophageal reflux disease (GERD) and OSA (Statement 30, Table 2), where GERD was considered a risk factor for most panelists but only regarded as a stated relationship for the others. More recently, a potential relationship between GERD and OSA was studied [37]. One mechanism suggested is that greater respiratory effort during the apnea and hypopnea events increases the pressure gradient across the lower esophageal sphincter (LES), facilitating the retrograde movement of gastric content [38].

More consistent disagreement (25%) was noted regarding the decreased risk of OSA in patients who had undergone previous tonsillectomy (Statement 32, Table 2). One explanation could be that some authors considered that performing the tonsillectomy in pediatric OSA patients could reduce the risk of OSA in adult age because of the positive impact on respiration improvement and maxillo-facial development [39].

Most of the disagreements in the section for patient criteria regarding the diagnosis of snoring and OSA were in the 21 sub-statements of 9 out of the overall 46 statements of this section. The most significant disagreement is the application of the Pittsburg sleep quality index questionnaire (PSQI), which is applied in daily routine only by 37.5% of the panelists (Table 2, Statement 17). This test was introduced in 1989 to analyze sleep quality in psychiatric patients and hereafter applied in OSA patients [40]. PSQI consists of 19 self-reported items and 5 additional questions for the bed partner regarding subjective sleep quality, sleep latency, sleep duration, habitual sleep efficiency, sleep disturbances, use of sleeping medication, and daytime dysfunction. Even though it has been translated into several European languages, its limited application among the panelists could be explained by the complexity of the test compared to easier ones, such the Epworth sleepiness scale (ESS) or stop-bang questionnaire, which allow a quicker assessment of the pathological daily somnolence or of the risk of being an OSA patient. ESS reached 100% agreement among the panelists (Statement 17, Table 2), confirming its application and usefulness in daily practice in patients with a suspicion of snoring and OSA [41,42].

In the third section, regarding the clinical evaluation of snoring and OSA, there was a high percentage of agreement (87.5–100%) in all the statements except for the usefulness of the Mueller maneuver (MM) for the OSA diagnosis (Statement 22, Table 3). Borowiecki and Sassin first described the MM for the preoperative assessment of OSA and it consists of a forced inspiratory effort against an obstructed airway with fiberoptic endoscopic visualization of the upper airway [43]. This maneuver is performed in awake patients, demanding an optimal ability by the patients to perform the inspiratory effort. Consequently, it is not helpful for analysis of the potential pattern of upper airway collapse in OSA patients while awake, as OSA is a sleep-related disorder. Another significant disagreement in this section regards drug-induced sleep endoscopy (DISE) and its usefulness for OSA diagnosis, only for 37.5% of the panelists (Statement 23, Table 3). The main controversies about DISE regard its impact on surgical outcomes [44,45] but there is an overall acceptance that the OSA diagnosis must be related only to a polysomnographic study assessment.

Finally, all the panelists concur in defining cephalometry, a CT scan, and MRI as unnecessary for the OSA patients’ assessment (Statements 19, 20, 21, Table 3).

The overall agreement among the panelists is significantly superior to the statements with disagreement. However, it has been concurred to leave the latter statements to highlight that unresolved issues are present in clinical and therapeutical OSA aspects.

Nevertheless, all the authors believed that the statements reported in the present study could increase the OSA knowledge among the practitioners, could help their training, as well as discussion amongst peers, and collaboration amongst other European Sleep Centers, reaching the future world level of acceptance in the majority of OSA issues. Furthermore, the disagreement level in specific statements may serve as a guide for research.

## 5. Conclusions

This consensus paper provides an overview of the results of an effort by a group of European experts in the field of sleep surgery to develop a set of statements on terminology and diagnostic processes related to snoring and OSA.

The varying levels of agreement and disagreement among experts, particularly regarding diagnostic methods, not only emphasize the multifaceted nature of OSA but also indicate key areas for future research and development in clinical practice, as well as the starting point for seeking consensus on treatment and outcomes.

## Figures and Tables

**Table 1:** Statements on the definitions regarding snoring and OSA.

Statements 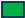 100% 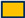 87.5% 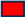 75% 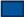 62.5% 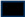 37.5%	% Consensus
1. Sleep is defined as a circadian state characterized by partial or total suspension of consciousness, voluntary muscle inhibition, and relative insensitivity to stimulation.	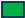 100
2. Snoring is defined as a rough rattling noise made by turbulent airflow mainly on inspiration during sleep caused by the vibration of, e.g., the soft palate, uvula, tonsils, tonsil pillars, the enlarged base of the tongue, epiglottis aryepiglottic folds, and/or arytenoids.	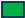 100
3. Sleep apnea is a potentially serious sleep disorder in which breathing repeatedly stops and starts.	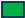 100
4. Sleep-disordered breathing (SDB) refers to a wide spectrum of sleep-related conditions including increased resistance to airflow through the upper airway, heavy snoring, marked reduction in airflow (hypopnea), and complete cessation of breathing (apnea).	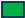 100
5. Obstructive sleep apnea syndrome (OSA) is a sleep disorder that involves cessation or a significant decrease in airflow in the presence of breathing effort.	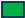 100
6. Obstructive sleep apnea syndrome is a decrease in the airflow at least 90%, occurs at least five times per hour, and lasts at least 10 s with the presence of respiratory efforts.	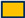 87.5
7. Hypopnea is defined as a reduction in ventilation of at least 30% that lasts at least 10 s which results in a decrease in arterial saturation of at least 3% or arousal.	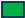 100
8. Mixed apnea is characterized by the absent respiratory effort and airflow in the first section of the event and respiratory effort without airflow in the last section (coexisting ventilatory control instability and upper airway collapsibility).	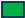 100
9. Apnea/hypopnea index (AHI) is the average frequency of apnea and hypopnea events per hour of sleep.	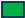 100
10. Positional sleep apnea (POSA) is defined as a total AHI ≥ 5 with at least 50% of respiratory events occurring in the supine position during sleep (AHI of at least twice as high in the supine position as a non-supine one.)	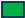 100
11. REM sleep is a phase of sleep with vivid dreams that happens about an hour to 1.5 h after falling asleep and is associated with greater sympathetic activity and cardiovascular instability than non-REM sleep. Patients who have REM-related OSA usually obtain worse results after sleep surgeries.	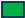 100
12. Non-REM sleep happens first and has three stages, the last two stages of non-REM sleep entail a deep sleep when it is hard to wake a person up.	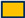 87.5
13. Sleep quality is defined as an individual’s self-satisfaction with all four attributes of sleep: sleep efficiency, sleep latency, duration, and wake after sleep onset.	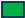 100
14. A sleep study is a test during sleep that measures specific sleep characteristics and helps to diagnose sleep disorders. There are four types of sleep study. For the diagnostic process and monitoring the results of the treatment in OSA, types I, II, and III of the sleep study are used.	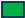 100
15. Polysomnography is a comprehensive test during sleep that records brain waves, the oxygen level in blood, heart rate, breathing, and eye and leg movements and also measures the sleeping position (at least seven physiological parameters). Type I is performed in the sleep lab and type II—at home.	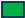 100
16. Polygraphy is a type III sleep study that records sleep parameters (at least four physiological parameters). Only some devices, like Watchpat, can measure neurophysiological parameters. It is generally not recommended for patients with cardiovascular disorders, pulmonary and neuromuscular diseases, or congestive heart failure.	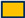 87.5
17. A home sleep study is performed at home, usually as a type III sleep study; however, a type II sleep study might be used as well.	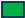 100

**Table 2:** Statements on the patient criteria regarding the diagnosis of snoring and OSA.

Statements	% Consensus
1. Snoring is an important health problem that may require treatment based on the severity and/or its impact on partners or other house members.	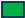 100
2. For diagnosis of snoring, history from members of the household/bed partner is	
a. Not sufficient	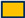 87.5
b. Not essential but can add important information	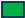 100
3. For diagnosis of snoring, audio/video recordings	
a. Are sufficient	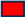 75
b. Are not essential but can add important information	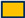 87.5
4. Objective methods (such as snoring apps) for the measurements of the loudness of primary snoring are useful but not necessary or essential.	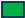 100
5. Subjective methods (such as the visual analog scale) for the loudness of primary snoring are useful but not necessary.	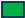 100
6. Subjective methods (such as the visual analog scale) for the loudness of primary snoring are not essential.	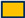 87.5
7. For the assessment of severity of snoring	
a. History from family members is not sufficient	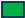 100
b. A Type 3 sleep study is not necessary	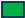 100
c. Polysomnogram is not necessary”	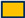 87.5
d. Audio/Video recording with mobile device is helpful	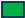 100
8. When there is snoring, sleep-disordered breathing should be suspected.	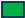 100
9. In order to rule out SDB or OSA when there is snoring, there is a need for a sleep study.	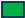 100
10. Sleep Questionnaires for the diagnosis of primary snoring are useful but not essential	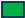 100
11. Sleep Questionnaires for the screening of OSA are useful but not necessary	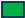 100
12. General (nonspecific to OSA) quality of life questionnaires are	
a. Useful	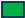 100
b. Disagree with “necessary or essential”	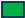 100
13. General (nonspecific to OSA) quality of life questionnaires, which I do not routinely use in my practice.	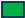 100
14. Specific to OSA quality of life questionnaires are	
a. useful	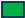 100
b. not essential	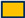 87.5
15. Specific to OSA quality of life questionnaires I routinely use in my practice	
a. PSQI	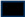 37.5
b. ESS	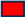 75
16. Stop-bang questionnaire for the diagnosis of primary snoring and OSA is	
a. Useful	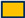 87.5
b. Not necessary	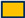 87.5
c. Not essential	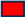 75
17. Epworth sleepiness scale questionnaire for the diagnosis of primary snoring and OSA is	
a. Useful	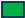 100
b. Not necessary	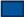 62.5
18. Pittsburgh sleep quality index questionnaire for the diagnosis of primary snoring and OSA is useful but not necessary or essential.	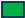 100
19. Glasgow benefit inventory questionnaire for the diagnosis of primary snoring and OSA is	
a. Useful	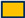 87.5
b. Not necessary or essential	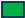 100
20. HADS questionnaire for the diagnosis of primary snoring and OSA is not necessary or essential.	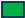 100
21. Daytime history elements to suspect OSA include tiredness after awakening, daytime sleepiness, morning headaches, falling asleep against an individual’s will, daytime fatigue, impairments in productivity and social functioning, and libido impairment.	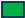 100
22. Nighttime history elements to suspect OSA include snoring, disruption of bed partner’s sleep, apneas observed by a bed partner, nocturnal choking, nocturia, gasping, awakenings with dry mouth, and insomnia.	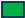 100
23. Workplace history elements to suspect.	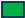 100
24. Car or workplace accident history to suspect.	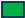 100
25. The presence of high BMI should raise suspicion for OSA.	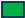 100
26. Presence of hypertension, diabetes, cardiovascular complications, hypothyroidism, and cognitive dysfunction may be related to OSA.	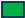 100
27. Use of alcohol should be seen as a risk factor for worse OSA.	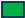 100
28. Age is a risk factor for snoring/OSA.	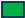 100
29. Presence of nasal allergies is an important risk factor for snoring/OSA.	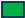 100
30. Presence of GERD is an important risk factor in OSA.	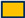 87.5
31. Presence of enlarged tonsils is an important risk factor in snoring/OSA.	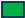 100
32. History of prior tonsillectomy does not reduce the risk for snoring/OSA.	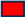 75
33. Presence of a long soft palate/large uvula are important risk factor in snoring/OSA.	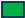 100
34. Presence of a large tongue is an important risk factor in snoring /OSA.	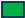 100

**Table 3:** Statements on the clinical evaluation of snoring and OSA.

Statements	% Consensus
1. A comprehensive history and physical examination are essential parts of the diagnosis of snoring/OSA.	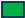 100
2. Flexible nasolaryngoscopy is a necessary part of the diagnostic evaluation of patients for snoring/OSA.	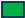 100
3. Assessment of mandibular position/occlusion and mandible protrusion is a necessary part of the physical examination.	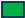 100
4. Assessment of tongue protrusion is a necessary part of the physical examination.	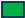 100
5. Grading the visualization of oropharyngeal visualization is a necessary part of the physical examination.	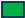 100
6. Assessment of the length of the soft palate is a necessary part of the examination.	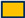 87.5
7. Assessment of the length and size of the uvula is a necessary part of the examination.	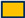 87.5
8. Assessment of neck size is a necessary part of the physical examination.	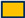 87.5
9. One-night polysomnography (type I or type II) for the diagnosis of OSA is	
a. Useful but not necessary	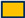 87.5
b. Not essential	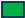 100
10. One night Type 3 sleep study (polygraphy/home sleep study) for the diagnosis of OSA is sufficient (polysomnography is not needed).	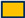 87.5
11. Two-night sleep study for the accurate diagnosis of OSA is useful but not necessary or essential.	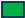 100
12. In the presence of cardiovascular co-morbidities for the diagnosis of SDB/OSA	
a. Type 3 sleep study (polygraphy/home sleep study) is sufficient	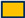 87.5
b. Type 3 sleep study (polygraphy/home sleep study) MAY BE sufficient	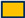 87.5
c. Polysomnography is NOT essential	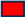 75
13. History and/or questionnaires are not sufficient for the diagnosis of OSA.	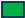 100
14. Criteria for a normal sleep study is AHI < 5, mean oxygen saturation 94–98%.	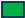 100
15. Criteria for mild OSA is 15 > AHI ≥ 5.	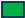 100
16. Criteria for moderate OSA is 30 > AHI ≥ 15.	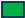 100
17. Criteria for severe OSA is ≥30.	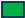 100
18. If 2-night sleep study is performed	
a. Average of the two nights is taken	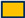 87.5
b. Second-night results are NOT more important	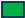 100
19. Cephalometry is not necessary in all patients.	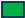 100
20. CT scan is not necessary in all patients.	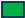 100
21. MRI is not necessary in all patients.	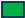 100
22. Mueller maneuver for the diagnosis of OSA is	
a. Useful	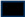 37.5
b. Not necessary	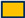 87.5
c. Not essential	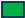 100
23 Drug-induced sleep endoscopy (DISE) for diagnosing the presence of OSA is	
a. Useful	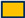 87.5
b. Not necessary	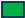 100
c. Not essential	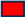 75
d. Not sufficient	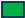 100
24. Drug-induced sleep endoscopy (DISE) for assessing the site (s) of obstruction in OSA is	
a. Useful	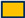 87.5
b. Not essential	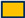 87.5
25. Detailed documentation of all the findings is an essential step in managing patients with snoring/OSA.	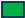 100
26. Evaluation of muscle tone strength is NOT an essential step in managing patients with OSA but may be useful.	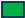 100

## Data Availability

Data are contained within the article.
